# HLA-A*02:07 Is a Protective Allele for EBV Negative and a Susceptibility Allele for EBV Positive Classical Hodgkin Lymphoma in China

**DOI:** 10.1371/journal.pone.0031865

**Published:** 2012-02-15

**Authors:** Xin Huang, Bouke Hepkema, Ilja Nolte, Kushi Kushekhar, Theo Jongsma, Rianne Veenstra, Sibrand Poppema, Zifen Gao, Lydia Visser, Arjan Diepstra, Anke van den Berg

**Affiliations:** 1 Department of Pathology and Medical Biology, University of Groningen, University Medical Center Groningen, Groningen, The Netherlands; 2 Department of Pathology, Health Science Center, Peking University, Beijing, China; 3 Department of Laboratory Medicine, University of Groningen, University Medical Center Groningen, Groningen, The Netherlands; 4 Unit of Genetic Epidemiology and Bioinformatics, Department of Epidemiology, University of Groningen, University Medical Center Groningen, Groningen, The Netherlands; University of Nebraska – Lincoln, United States of America

## Abstract

HLA-A2 protects from EBV+ classical Hodgkin lymphoma (cHL) in Western Europe, but it is unknown whether this protective effect also exists in the Chinese population. We investigated the association of HLA-A2 and specific common and well documented HLA-A2 subtypes with EBV stratified cHL patients (n = 161) from the northern part of China. Quantitative-PCR and sequence-based subtyping was performed to identify HLA-A2 positive samples and their subtypes. 67 (42%) of the cHL patients were EBV+. There were no significant differences in percentages of HLA-A2 positivity between cHL and controls (65% vs 66%) and between EBV+ and EBV− cHL patients (70% vs 61%). The frequency distribution of HLA-A2 subtypes was significantly different between EBV stratified cHL subgroups and controls. This difference was most striking for the HLA-A*02:07 type with a frequency of 38% in EBV+ cHL, 8% in EBV− cHL and 20% in controls. Significant differences were also observed for the HLA-A*02:07, HLA-A2 (non-02:07) and the A2-negative typings between EBV+ cHL vs controls (p = 0.028), EBV− cHL vs controls (p = 0.045) and EBV+ vs EBV− cHL cases (p = 2×10^−5^). In conclusion, HLA-A*02:07 is a predisposing allele for EBV+ cHL and a protective allele for EBV− cHL in the northern Chinese population.

## Introduction

Classical Hodgkin lymphoma (cHL) has a complex multi-factorial etiology with both genetic and environmental factors contributing significantly to its development [1], [2]. Epstein-Barr virus (EBV), a ubiquitous human gamma-herpes virus that infects over 90% of the population worldwide, has been consistently linked to the pathogenesis of a subset of cHL [1], [3]. Epidemiological data indicates a bimodal disease model with different pathogenetic backgrounds for EBV+ and EBV− cHL. Despite its strong oncogenic potential, only a minority of EBV-infected individuals develop EBV+ cHL [3], because of effective anti-viral immune responses.

The incidence of EBV+ cHL is strikingly increased in immunocompromised patients [4] indicating the importance of an effective immune system in controlling the EBV+ cell population. The human leukocyte antigen (HLA) is a crucial component of the human immune system and HLA class I restricted, CD8+ cytotoxic T-cell (CTL) responses are known to play a pivotal role in the control of EBV infection [5]. In EBV+ cHL there is a so-called latency type II expression of viral genes by the tumor cells that is restricted to the two latent membrane protein antigens (LMP1 and LMP2) and EBV nuclear antigen 1 (EBNA1). Induction of LMP/EBNA1-specific immune responses has been successful in the context of appropriate HLA molecules [6]–[10].

Genetic association between HLA and cHL has been investigated in the Dutch, British and Scandinavian population and HLA-A2 has consistently been reported as a protective type for developing EBV+ cHL, while HLA-A1 is considered a risk type [11], [12]. A tentative explanation for this association is the known presence of multiple cytotoxic T cell epitopes for LMP2 derived antigenic peptides restricted through HLA-A2, whereas HLA-A1 restricted epitopes to the latent EBV peptides have not been found [7], [13]. HLA-A2 has a high prevalence worldwide [14] and is one of the most diverse allele families of the HLA-A locus consisting of over 300 allelic variants (IMGT/HLA database v 3.3.0). These alleles show a marked difference in the capacity of peptide binding and presentation [15], [16] even for alleles that differ by only one amino acid in the peptide binding groove. For example, presentation of EBV derived peptides by the HLA-A*02:01 allele induces stronger immune responses as compared to other HLA-A2 alleles, including HLA-A*02:07 [8]. The distribution of these HLA-A2 allelic variants differs widely by geography and ethnicity [14]. In the Caucasian population, >90% of the HLA-A2 positive individuals carry the HLA-A*02:01 allele, whereas in the Chinese population there are multiple common and well-documented (CWD) allelic variants, including HLA-A*02:01, A*02:03, A*02:06, A*02:07 and A*02:10 [14], [17]. Based on these marked differences, it can be postulated that interracial variations influence the host immune response and this may lead to different HLA-A associations in different populations.

Consistent with the Dutch population, HLA class I cell surface expression was observed in the tumor cells of the vast majority of Chinese EBV+ cHL patients [18]. In this study, we determined the HLA-A2 phenotype frequency in Chinese cHL patients as well as in controls from the same geographic region. The CWD HLA-A2 allelic variants were determined by sequence-based typing (SBT).

## Results

### HLA-A2 carrier frequency

**Figure 1:**
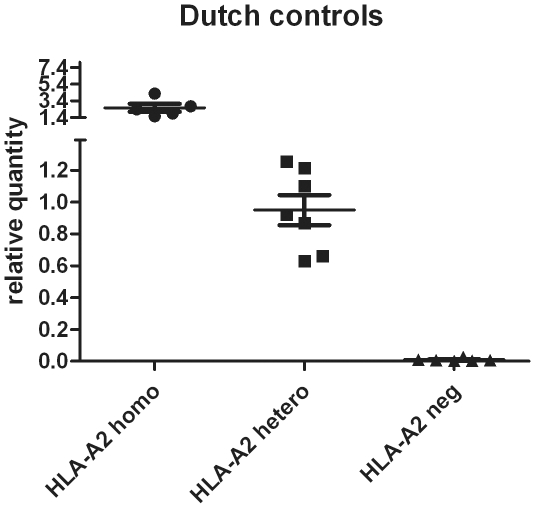
Validation of the HLA-A2 specific primer set on DNA samples isolated from FFPE tissue sections from individuals with known HLA genotypes. A clear difference can be observed between samples possessing two or one HLA-A2 allele(s) and those without HLA-A2 alleles. Homo, homozygous; hetero, heterozygous, neg, negative.

Analysis of the 21 FFPE tissue samples of cHL patients with known HLA-A genotype revealed a clear separation between individuals lacking HLA-A2 alleles and individuals heterozygous or homozygous for the HLA-A2 type (Figure 1). The relative expression values varied between 0.002 and 0.027 for negative patients and between 0.63 and 4.26 for positive patients.

The HLA-A2 carrier frequency in the total cHL patient group (104 out of 161, 65%) was similar to the control group (79 out of 119, 66%). In contrast to the Western population, no difference was observed between the HLA-A2 frequency in EBV+ (47 out of 67, 70%) and EBV− Chinese cHL patients (57 out of 94, 61%) (Table S1). This might be due to differences in HLA-A2 allelic variants in the Chinese compared to the Dutch and Caucasian populations.

### HLA-A*02:07 risk allele analysis

HLA-A2 subtyping failed in 5 cHL patients and 3 controls due to inefficient amplification of both exons and/or poor sequencing results. For part of the patients subtyping was based only on the exon 3 sequence analyses, which did not allow discrimination between all of the CWD alleles. SBT results of the 99 HLA-A2 positive cHL patients and the 76 positive controls are shown in Table S2. The distribution of HLA-A2 subtypes in controls and the total cHL patient group was similar. In EBV status defined subgroups the allele frequency for HLA-A*02:07 showed marked differences, i.e. higher in EBV+ cHL and lower in EBV− cHL patients. Analysis of the frequency distribution of HLA-A2 negative, HLA-A*02:07 positive and HLA-A2 (non A*02:07) individuals revealed significant differences between controls and EBV status defined subgroups of cHL patients and also between the EBV+ and EBV− cHL groups (Table 1).

**Table 1:** HLA-A*02:07 carrier frequency in Chinese controls and cHL patients.

HLA type	ControlsN (%)	all cHL N (%)	EBV+ cHL N (%)	Controls vs EBV+ p-value^I^	EBV− cHL N (%)	Controls vs EBV− p-value^I^	EBV+ vs EBV− p-value^I^
A*02:07	23 (19.8%)	31 (19.9%)	24 (37.5%)	**0.028**	7 (7.6%)	**0.045**	**2×10^−5^**
A2 (non A*02:07)	53 (45.7%)	68 (43.6%)	20 (31.3%)		48 (52.2%)		
A2 negative	40 (34.5%)	57 (36.5%)	20 (31.3%)		37 (40.2%)		
Failure*	3	5	3		2		

I Chi-square test comparing A*02:07, A2 (non A*02:07) and A2 negative frequencies between groups as indicated. There were no significant differences between controls and the total cHL group.

*The failures are A2 positive patients (in table S2) for whom the PCR for SBT failed due to poor quality DNA.

**Figure 2:**
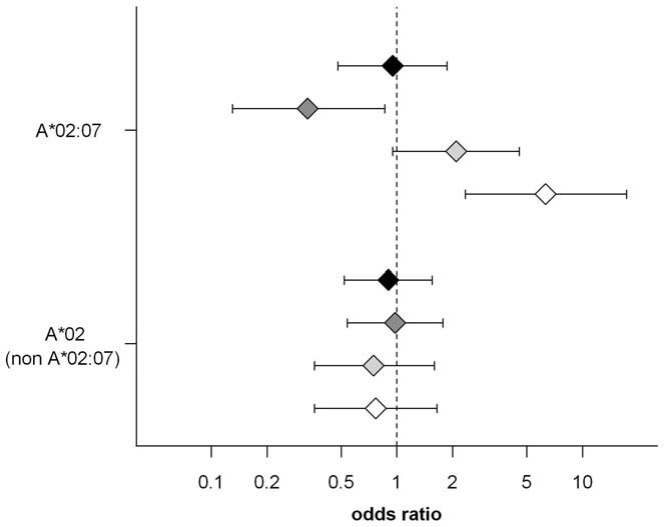
Odds ratios for HLA-A*02:07 subgroups for all cHL patients versus controls (black), EBV negative cHL patients versus controls (dark grey, p = 0.023), EBV positive cHL patients versus controls (light grey, p = 0.066) and EBV positive versus EBV negative cHL patients (white, p = 0.0003). The HLA-A*02 negative individuals constitute the reference group. Bars indicate the 99% confidence intervals.

The odds ratio (OR) for HLA-A*02:07 was significantly increased in EBV+ cHL as compared to EBV− cHL (OR = 6.34, 95% CI = 2.33–17.28, p = 0.0003). In comparison to controls the OR of EBV+ cHL was 2.09 (95% CI = 0.95–4.57, p = 0.066) and the OR for EBV− cHL was 0.33 (CI 0.13–0.86, p = 0.023) for HLA-A*02:07. No differences were observed for EBV stratified cHL subgroups in comparison to controls for the HLA-A2 (non A*02:07) type (Figure 2).

Multivariate analysis revealed that both mixed cellularity subtype and HLA-A*02:07 positivity were significantly associated with positive EBV status. Age, sex and HLA-A*02:xx (non A*02:07) were not significantly associated. In our analysis HLA-A*0207 status remained significant even after adjusting for age, sex and diagnosis.

## Discussion

HLA-A2 has been identified as a protective type and HLA-A1 as a risk type for the development of EBV+ cHL in the Dutch, British and Scandinavian population [11], [12]. In this study, we found no difference of the HLA-A2 carrier frequency between EBV status defined cHL patient groups and controls from Northern China. However, a more detailed typing of specific CWD HLA-A2 alleles in the Chinese population revealed that HLA-A*02:07 is a risk allele for EBV+ cHL, whereas this allele is protective for EBV− cHL.

Our approach, using combined analyses of HLA-A2 specific qPCR and sequence-based typing of the CWD subtypes, was of crucial importance for identification of risk and protective alleles in the Chinese population. Moreover, this study illustrates the importance of considering differences in HLA subtype prevalence in ethnic groups in genetic association studies especially for associations in the HLA region. Differences in HLA types might also explain the striking differences in cHL incidence in different racial groups with a nearly 6-fold difference between Western Europe and East Asia with an incidence of 2.3 and 0.4 per 100,000 inhabitants per year respectively in 2008 (http://globocan.iarc.fr/).

We observed similar HLA-A2 frequencies in EBV+ and EBV− Chinese cHL patients, whereas suballele frequencies differed significantly with an increased percentage of HLA-A*02:07 in EBV+ patients and a reduced frequency in EBV− cHL patients. Multivariate analysis indicated that both histological subtype and HLA-A*02:07 were significantly associated with EBV, whereas age and sex were not. Thus, our data indicate that the association of HLA-A*02:07 with EBV status defined subgroups remained significant after adjusting for age, sex and subtype.

HLA-A*02:07 is almost exclusively present in the eastern Asian population and is the second most common allele just after the HLA-A*02:01 allele [14]. This is also true for the northern Chinese population, while in southern China the HLA-A*02:07 allele frequency was reported to be even higher than the HLA-A*02:01 frequency [17]. The single difference between the HLA-A*02:01 allele and the HLA-A*02:07 allele at the protein level is a unique amino acid change (Y_99_ to C). The question arises if this single amino acid difference can indeed make a difference between a risk and a protective allele. It has been shown that the peptide binding repertoire of HLA-A*02:07 is limited to a subset of the peptides presented by HLA-A*02:01 [8], [10], [19], [20] or includes a distinct set of peptides that were not presented by other HLA-A2 alleles [16]. Khanna et al showed that LMP1 peptides induced a cytotoxic T cell response against EBV-infected B cells in the context of several HLA-A2 alleles, including HLA-A*02:01, HLA-A*02:02, HLA-A*02:03, HLA-A*02:04 and HLA-A*02:06 [21]. Lee et al demonstrated a less effective response to LMP2 peptides using cytotoxic T cells from individuals carrying HLA-A*02:07 as compared to individuals carrying HLA-*02:01, HLA-A*02:06 or HLA-A*02:09 [8].

In the Dutch, British and Scandinavian population HLA-A1 was shown to be a risk allele for developing EBV+ cHL [11], [12]. This allele is fairly uncommon in the Chinese population and we did not observe significant differences in HLA-A1 carrier frequencies between controls and EBV stratified cHL patients using an HLA-A1 specific qPCR approach (results not shown). On the amino acid level there are many (n = 33) differences between the HLA-A*01:01 allele and the HLA-A*02:07 allele, including Y_99_ (is C in HLA-A*01:01). Thus, both susceptibility alleles differ largely, while the protective HLA-A*02:01 allele differs from HLA-A*02:07 by only a single amino acid (Y_99_ to C) as discussed above. It remains unclear what the functional overlap between the two highly different HLA-A*01:01 and HLA-A*02:07 alleles is, but the consensus is that both alleles are ineffective in the production of an interferon-gamma response against LMP1 or LMP2 peptides [7], [8], [13]. An explanation for the risk of developing EBV+ cHL linked to the identified alleles might thus be found in differential specificity of binding and presentation of antigenic peptides.

The HLA-A*02:07 allele is also associated with an increased risk of undifferentiated nasopharyngeal carcinoma (UNPC) in the southern Chinese population [22]. UNPC is an EBV-associated malignancy and has an identical latency type of infection as cHL with an expression pattern that is restricted to LMP1, LMP2 and EBNA1 [23]. In the Caucasian population in the US HLA-A*02:01 was associated with a decreased risk for UNPC [24], [25]. The associations are similar to our findings in cHL, although HLA-A1 is not a known susceptibility type for developing UNPC in the Chinese population. Combination of our data showing an association specifically in the EBV+ subgroup to the findings in UNPC makes it now possible to more firmly link the HLA-A*02:07 association directly to the ineffectiveness of this allele to present LMP1 and LMP2 derived antigenic peptides in UNPC.

An unexpected finding in our study was the significantly decreased HLA-A*02:07 frequency in the EBV- cHL population as compared to controls. In theory, this might be explained by differences in the composition of the pool of endogenous (mutated) proteins. Thus, HLA-A*02:07 might induce strong immune responses against peptides that are non-EBV related and that are predominantly present in EBV− cHL. This effect should probably take place early in disease pathogenesis, as HLA class I expression by EBV− cHL tumor cells is frequently downregulated in cHL patients at the time of diagnosis, also in Chinese cHL cases [18].

In conclusion, our study reports for the first time that the HLA-A*02:07 allele has a strong predisposing effect for EBV+ cHL in the Chinese population, while this allele is protective for EBV− cHL. Discrimination between subtypes of HLA-A2 was of central importance for understanding the genetic association of HLA-A2 with EBV+ cHL. Differences in HLA subtype frequencies in different populations provide a unique opportunity for further understanding the influence of genetic heterogeneity in HLA and its role in the pathogenesis of EBV+ cHL.

## Materials and Methods

### Study Population

Formalin-fixed paraffin-embedded (FFPE) tissue blocks of lymph node biopsies from 161 cHL patients obtained from 5 hospitals in northern China (Dept. of Pathology, Health Science Center, Peking University; Dept. of Pathology, First Hospital of Jilin University; Dept. of Pathology, Shougang Hospital, Peking University; Dept. of Pathology, Beijing Air Army General Hospital; Zhanye Regional Hospital, Gansu Province) were used for this study. Results of the hematoxylin & eosin (H&E) staining, reclassification according to the WHO classification of 2008 and EBER in situ hybridization (ISH) have been published previously [18]. 67 of the 161 cHL were positive for EBV (42%).

To minimize genetic variations we included a control group of 119 reactive lymph nodes with lymphoid reactive hyperplasia (n = 88) or Kikuchi disease (n = 31) obtained during the same period from the Dept. of Pathology, Health Science Center, Peking University as a control group. All analysis were done with and without the Kikuchi controls and revealed the same allele frequencies.

FFPE tissue blocks of 21 cHL patients with known HLA genotype were retrieved from the University Medical Center Groningen (UMCG), the Netherlands. These samples were used to optimize and validate the HLA-A2 specific quantitative (q)PCR and included 6 A2-homozygotes, 7 A2-heterozygotes and 8 non-A2 genotypes. All procedures were carried out according to institutional ethical review board guidelines of the UMCG. Research was conducted adhering to the Declaration of Helsinki and according to Dutch regulations (http://www.federa.org/). All tissue samples were anonymyzed before use.

### DNA isolation

2∼3 10 µm tissue sections were incubated overnight at 55°C in 242 µL of PK1 lysis buffer (10 mM Tris pH 8.0, 50 mM KCl, 2.5 mM MgCl2, 0.45% NP40, 0.45% Tween 20, 0.01% gelatin) with 7.5 µL Proteinase K (20 mg/ml, Invitrogen). Proteinase K was heat inactivated for 5 min at 100°C. The supernatant containing the DNA was carefully transferred into a new tube. DNA was quantified by nanodrop-1000 spectrophotometer and diluted to a concentration of 5 and 10 ng/µL for the subsequent PCR analysis.

### HLA-A2 quantitative (q)PCR

qPCR assays were carried out on an ABI PRISM 7900HT (Applied Biosystems) with SYBR-green in a 384-well microtiter plate using primers specific for HLA-A2 (Table S3). As a control for quality and quantity we used a primer set for the PTP4A1 gene. All reactions were performed in triplicate in a final reaction volume of 20 µl consisting of 10 µl SYBR® Green PCR master mix (Applied Biosystems), 2 µl (3 mM) of each primer and 5 µl (5 ng/µl) DNA. Each sample was analyzed in triplicate, starting with a 2-min AmpErase UNG activation step at 50°C and a 10-min hot start at 95°C, followed by 45 cycles of denaturation at 95°C for 15 s and combined annealing/extension at 60°C for 1 min. A melting curve was generated (95°C for 15 s, 60°C for 15 s, and 95°C for 15 s) to verify specificity of the PCR products. Cycle threshold (Ct) values were determined by using the default baseline setting of 3 to 15 cycles. All data were analyzed by using ABI PRISM 7900 HT Sequence Detector Systems software version 2.3 (Applied Biosystems). Samples with poor DNA quality were excluded based on lack of a specific peak in the melting curve or a Ct value higher than 36 for PTP4A1 (5 controls and 24 patients were excluded). HLA-A2 Ct values were normalized against the Ct value for PTP4A1, resulting in a delta Ct. Relative abundance of HLA-A2 was calculated by using the formula 2^−ΔCt^. Based on the 21 previously HLA-A typed samples, the cut-off level for HLA-A2 positivity was set at a 2^−ΔCt^ value of 0.1. A 2^−ΔCt^ value of 0.05 or less was considered to indicate absence of HLA-A2.

### HLA-A2 subtypes

Subtyping of HLA-A2 positive individuals was performed by amplification and sequence analysis of exon 2 and exon 3 regions that contain a number of SNPs that discriminate between the common and well-documented (CWD) allelic variants in the Chinese population (Table 1, Figure S1). DNA samples were amplified in a volume of 25 µl containing 5 µl (10 ng/µl) DNA, 5 µl (2 mM) of each primer, 1.25 µl dNTPs (Invitrogen), 2.5 µl 10× reaction buffer (Invitrogen), 5.85 µl H_2_O and 0.4 µl Taq Polymerase (Invitrogen). Amplification was performed in a GeneAmp PCR System 9700 thermocycler (Applied Biosystems) with an initial 3-min hold at 95°C and 40 cycles of 15 s at 95°C, 30 s at 65°C, and 1 min at 68°C, followed by a final 10 min extension step at 68°C. PCR products were visualized on a 1% agarose gel and purified by Exo-SAP treatment (USB products, Affymetyrics, Cleveland, Ohio USA). PCR products were sequenced using the same primers as the ones used for amplification. Due to the longer amplicon size of the exon 2 PCR products a number of samples failed for the analysis of the SNPs in this region leading to a less effective discrimination between the CWD HLA-A2 subtypes in these samples. This led to ambiguities for some alleles. Using the primer set as indicated above we were not able to discriminate between HLA-A*02:01 and HLA-A*02:03, leading to a HLA-A*02:01/02:03 ambiguity. Sequence data were analyzed using Seqman software (DNA Star, Madison, WI).

### Statistical Analysis

Significant differences in HLA-A2 positivity and in phenotype frequency of HLA-A2 subtypes between the total cHL group and the control subjects as well as those between EBV+ and EBV− cHL patients were assessed by Chi-square. Logistic regression analyses were used to determine the odds ratios and their 95% confidence intervals. A p-value<0.05 was considered significant. A multivariate analysis was done by logistic regression analysis using EBV status as the dependent variable and age (continuous), sex, diagnosis and HLA-A*02 status as independent variables. The analyses were performed in PASW Statistics version 18 (SPSS Inc., Chicago, Illinois, USA) and Microsoft Excel® 2003 (Microsoft corporation, Redmond, Washington, USA).

## Supporting Information

Figure S1 **Alignment of the HLA-A gene fragments that are amplified and sequenced to discriminate between common and well defined HLA-A2 alleles.** The five SNPs that are able to discriminate between the major HLA-A2 subtypes (IMGT/HLA database, http://www.ebi.ac.uk/imgt/hla/) are indicated by boxes. (**A**) Two SNPs located at position 98 and 102 are specific for HLA-A*02:05, A*02:06 and A*02:10. (**B**) SNPs located at position 355 and 402 are specific for HLA-A*02:05 only and one SNP at position 368 can differentiate both HLA-A*02:07 and A*02:10 from others. Numbers above the sequences indicate nucleotide positions. Nucleotide sequences were aligned to the HLA-A*01:01 allele. Dashes indicate identity with the reference sequence. Supplementary information is available at Genes and Immunity's website http://www.nature.com/gene/index.html.
(DOC)

Table S1 **Phenotype frequencies of HLA-A2 in Chinese cHL patients and controls.**
(DOC)

Table S2 **CWD HLA-A2 alleles in HLA-A2 positive controls and cHL patients.**
(DOC)

Table S3 **Primer sequences for allelic discrimination.**
(DOC)
