# Supplementary material for: HLA-A*02:07 Is a Protective Allele for EBV Negative and a Susceptibility Allele for EBV Positive Classical Hodgkin Lymphoma in China
Source: PLoS One. 2012 Feb 15;7(2):e31865. doi: 10.1371/journal.pone.0031865 (PMC3280205; doi:10.1371/journal.pone.0031865)
Supplement: Table S1 — Phenotype frequencies of HLA-A2 in Chinese cHL patients and controls. (DOC) [file pone.0031865.s002.doc]

**Supplementary Table S2** Phenotype frequencies of HLA-A2 in Chinese cHL patients and controls

|  | **Controls***  N=119 (%) |  | **All patients***  N=161 (%) |  | **EBV+ cHL***  N=67 (%) |  | **EBV- cHL***  N=94 (%) |  |
| --- | --- | --- | --- | --- | --- | --- | --- | --- |
|  |  |  |  |  |
| **HLA-A2-pos** | 79 (66.4) |  | 104 (64.6) |  | 47 (70.1) |  | 57 (60.6) |  |
| **HLA-A2-neg** | 40 (33.6) |  | 57 (33.4) |  | 20 (29.9) |  | 37 (39.4) |  |

*There were no significant differences for controls vs patients, EBV+ vs controls and EBV- vs controls and EBV+ vs EBV- cHL patients.
