# Supplementary material for: HLA-A*02:07 Is a Protective Allele for EBV Negative and a Susceptibility Allele for EBV Positive Classical Hodgkin Lymphoma in China
Source: PLoS One. 2012 Feb 15;7(2):e31865. doi: 10.1371/journal.pone.0031865 (PMC3280205; doi:10.1371/journal.pone.0031865)
Supplement: Table S2 — CWD HLA-A2 alleles in HLA-A2 positive controls and cHL patients. (DOC) [file pone.0031865.s003.doc]

**Supplementary Table S3** Common well documented HLA-A2 alleles in HLA-A2 positive controls and cHL patients

|  | **76 controls**  **n=83 (%)** | **99 cHL**  **n=107 (%)** | **44 EBV+ cHL**  **n=50 (%)** | **55 EBV- cHL**  **n=57 (%)** |
| --- | --- | --- | --- | --- |
| **HLA-A2 alleles^a^** |
| **A*02:01/02:03** | 27 (33.8%) | 31 (30.4%) | 9 (19.1%) | 22 (40.0%) |
| **A*02:01/02:03/02:06** | 17 (21.3%) | 16 (15.7%) | 7 (14.9%) | 9 (16.4%) |
| **A*02:01/02:03/02:05/02:06** | 2 (2.5%) | 0 (0.0%) | 0 (0.0%) | 0 (0.0%) |
| **A*02:05** | 1 (1.3%) | 1 (1.0%) | 0 (0.0%) | 1 (1.8%) |
| **A*02:06** | 11 (13.8%) | 25 (24.5%) | 9 (19.1%) | 16 (29.1%) |
| **A*02:05/02:06/02:10** | 1 (1.3%) | 0 (0.0%) | 0 (0.0%) | 0 (0.0%) |
| **A*02:07** | 23 (28.8%) | 31 (30.4%) | 24 (51.1%) | 7 (12.7%) |
| **A*02:10** | 1 (1.3%) | 3 (2.9%) | 1 (2.1%) | 2 (3.6%) |
| **failure** | 3 (3.6%) | 5 (4.7%) | 3 (6.0%) | 2 (3.5%) |

^a^Eight patients and seven controls showed a heterozygous pattern for two HLA-A2 alleles resulting in a total of 83 (76+7) A2 alleles for the controls and 107 (99+8) A2 alleles for the cHL patients. Five patients and one control were heterozygous for HLA-A*02:03 and HLA-A*02:07, two patients and one control were heterozygous for HLA-A*02:06 and HLA-A*02:07, one patient was heterozygous for HLA-A*02:06 and HLA-A*02:10, one control was heterozygous for HLA-A*02:05 and HLA-A*02:07, one control for HLA-A*02:01/02:03/02:06 and HLA-A*02:07 and three controls were heterozygous for HLA-A*02:01/02:03 and HLA-A*02:06.
