# Supplementary material for: HLA-A*02:07 Is a Protective Allele for EBV Negative and a Susceptibility Allele for EBV Positive Classical Hodgkin Lymphoma in China
Source: PLoS One. 2012 Feb 15;7(2):e31865. doi: 10.1371/journal.pone.0031865 (PMC3280205; doi:10.1371/journal.pone.0031865)
Supplement: Table S3 — Primer sequences for allelic discrimination. (DOC) [file pone.0031865.s004.doc]

**Supplementary Table S1** Primer sequences for allelic discrimination

| **Allele specificity** | **Primer sequence* (5’ – 3’)** | **Location** | **Product size** |
| --- | --- | --- | --- |
| **HLA-A2** | F: GAGCCCCGCTTCATCGC**A** | Exon 2 | 150bp |
|  | R: C**CC**GTCCCAATACTCCGG**A** | Exon 2 |  |
| **PTP4A1** | F: GCACAGCACGACCTCTATGC | Exon 2 | 142bp |
|  | R: CCAGGTCAGAACTCTTGTAAAATGC | Exon 2 |  |
| **HLA-A2 exon 2** | F: CTCTG**T**GGGGAGAAGCAAC | Intron 1 | 191bp |
|  | R: GTCGTCCACGTAGCCCAC**T** | Exon 2 |  |
| **HLA-A2 exon 3** | F: GCGGGGCTCGGGGGAC**C** | Intron 2 | 131bp |
|  | R: GCCGTCGTAGGCGT**A**CTG**G** | Exon 3 |  |

*F, forward primer; R, reverse primer. Nucleotides in bold indicate the positions of the SNPs specific for the CWD HLA-A2 alleles.
